# Effect of Socks on the Assessment of Vibration Sensation

**DOI:** 10.1155/2013/327960

**Published:** 2013-10-24

**Authors:** R. C. Meral, Z. Matur, B. Dertsiz, A. E. Öge

**Affiliations:** ^1^Istanbul Faculty of Medicine, Istanbul University, Capa, 34093 Istanbul, Turkey; ^2^Department of Neurology, Istanbul Faculty of Medicine, Istanbul University, Capa, 34093 Istanbul, Turkey

## Abstract

*Objective*. To investigate the difference between the measurement of vibration sensation without and with socks. *Material and Methods*. Fifty healthy volunteers (H group) and 19 patients with diabetic polyneuropathy (PNP group) were included. The sites of measurement were the great toe (GT) and medial malleolus (MM). A standard 128 Hz tuning fork was used in the measurements. *Results*. Mean duration of vibration sensations without and with socks was as follows: in the H group, 19.4 ± 4.2 and 19.5 ± 4.2 s at GT and 15.1 ± 3.3 and 14.6 ± 3.3 s at MM; in the PNP group, 13.4 ± 3.8 and 12.7 ± 4.1 s at GT and 11.9 ± 3.8 and 11.7 ± 3.4 s at MM. No significant difference was found between the measurements without and with socks, except those found at the MM in group H (*P* = 0.02). This significant difference was further analyzed in terms of effect size which was concluded to be practically insignificant (Cohen's *d* < 0.2). Shorter mean vibration duration was measured at MM as compared to GT that could be explained by the damping effect. *Conclusions*. Wearing socks of moderate thickness does not have any important effect on the duration of vibration sensation. This might be considered as a reflection of the remarkable properties of vibration sensation.

## 1. Introduction

The evaluation of vibration sensation informs the clinician about the integrity of mechanoreceptors in the skin, rapidly conducting large-diameter afferent fibers in the peripheral nerves and the dorsal column-medial lemniscus pathways in the central nervous system [1, 2]. Therefore, diminished vibration sensation is an important finding in the diagnosis of disorders affecting the dorsal column-medial lemniscal system and may also be an early sign of peripheral neuropathies [3]. It is known in everyday practice that patients, due to the physical properties of this sensory modality, can perceive vibration through layers of clothes [4]. However, the validity of examining vibration sensation over the clothes is unknown. This recently resulted in a debate during a case presentation in our clinic in which one of the authors tried to perform the examination on a clothed subject for the sake of speed. The present study was designed under the inspiration of this dispute with the hope of providing some new insights into the characteristics of vibration sensation.

## 2. Methods

### 2.1. Subjects

Fifty healthy volunteers (H group; median age was 37 years, 22 male) and 19 patients with diabetic polyneuropathy (PNP group; median age was 57 years, 5 male) were included in the study (Table 1). The H group mainly consisted of volunteer relatives of patients, healthcare professionals, and medical students stratified into 4 age groups, as follows: ages 18–25 (*n* = 14), 26–40 (*n* = 13), 41–55 (*n* = 13), and 55+ (*n* = 10). Their mean height was 168.5 ± 9.1 cm (range 153 to 183) and their mean weight was 72.2 ± 14.6 kg (range 49 to 110). A brief neurological examination including superficial sensation, muscle strength, and tendon reflexes was made and found to be normal in all the subjects in this group. Those who had any neurological and systemic disease or who were using medications which could affect the peripheral or central nervous systems were excluded from the study.

The PNP group consisted of patients with known type 2 diabetes mellitus and the clinical diagnosis of diabetic polyneuropathy visiting the diabetic outpatient clinic for their regular controls (median follow-up duration was 14 years; range 1–26).

### 2.2. Measurement of the Vibration Sensation

Vibration sensation was measured with the common psychophysical technique, whereby the duration of vibration sensation is dependent on each subject's judgment. The subjects lay supine in a comfortable quiet room with an ambient temperature of 20°–25°C during the measurements. A 128 Hz standard tuning fork was used to assess the vibration sensation throughout the study (Riester Tuning Fork w/clamps, Aluminum, no. 5162, C 128 Hz, California, USA). The socks used in this study all had the same thickness and tissue characteristics (the same model from a certain manufacturer; cotton, 70 denier linear mass density) which had been purchased in bulk and were disposed of after a single use. 

**Figure 1:**
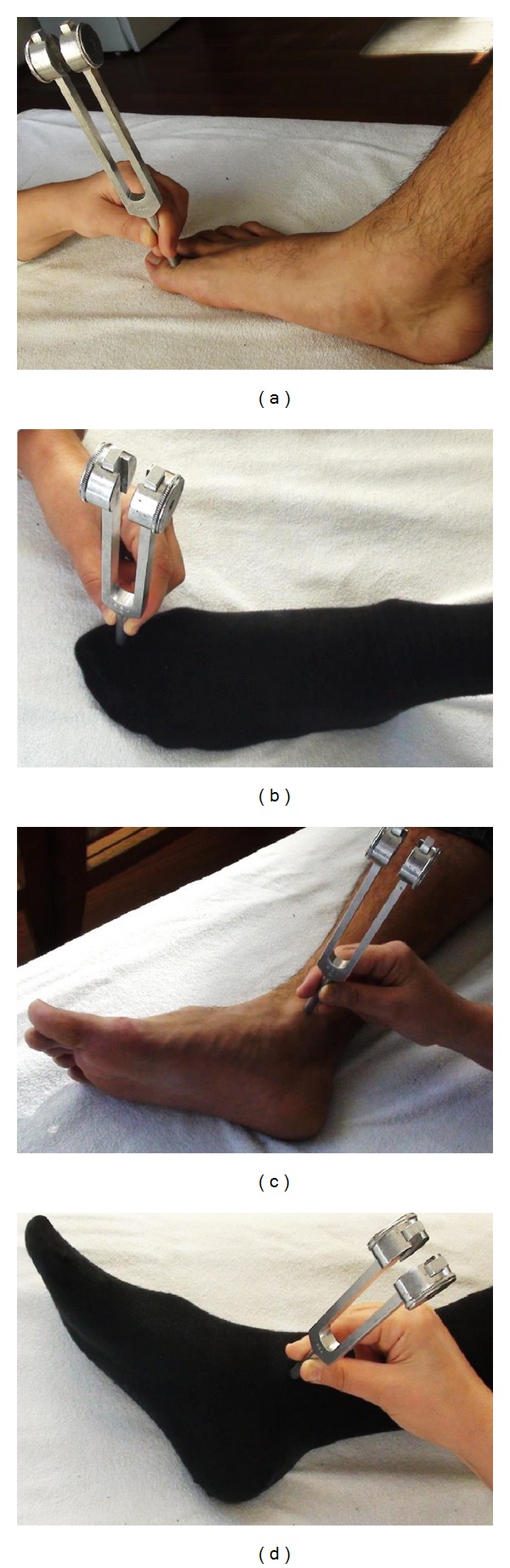
Measurement technique. (a), (b): Measurement at the great toe without and with socks. (c), (d): Measurement at the medial malleolus without and with socks.

The positions of the subjects' feet during measurements are shown in Figure 1. Four sites were selected for measurements: dorsum of the interphalangeal joint of the great toe (GT) and the vertex of the medial malleolus (MM) on both sides. The tuning fork was applied vertically to these points with gentle pressure after the prongs were maximally activated (by tapping the prongs against the examiner's hypothenar eminence) for each measurement (Figure 1). Two investigators took the measurements; the first one applied the tuning fork while the second investigator measured with a stopwatch the duration of vibration sensation from the activation time of the tuning fork until the subject's verbal indication that the sensation was over.

Initially, the procedure was rehearsed and subjects were taught about vibration sensation. Those who were found to have no vibration sensation at all on any of the measurement sites (usually at GT) were excluded from the study at this point. In the measurement phase, 2 measurements without and 2 measurements with socks were performed on each site in the following sequence, allowing a five-minute break inserted so as not to exhaust the subjects and to help them maintain their concentration: without, with, interval, with, without. Sixteen measurements in total were taken for each subject, requiring about 20 minutes for the entire procedure. The averages of each pair of measurements with and without socks were used as the statistical input. 

The procedure was approved by the Istanbul Faculty of Medicine Ethics Committee (2012/1244) and all participants provided informed consent.

### 2.3. Data Analysis

All the statistical analyses were performed using the Statistical Package for the Social Sciences (SPSS), version 17.0, for Windows (SPSS, Chicago, IL, USA).

1. Samples were tested for their distribution with Kolmogorov-Smirnov test.
2. Correlations between the measurements in the same conditions (first and second measurements on the same side without socks and with socks) and between the measurements on both sides were tested with Pearson's *R*.
3. After showing a high correlation between the items in [2], the results of the two measurements for each condition and the results elicited on the right and left sides were averaged to constitute the main inputs for the statistical analyses.
4. The difference between the mean durations of vibration sensation without and with socks in both groups was analyzed using paired samples *t*-test.
5. Statistically significant results elicited in [4] were analyzed for the effect size, using Cohen's *d*.
6. In the H group, correlations of vibration sensation with age, height, and weight were assessed with Pearson's *R*.
7. Independent samples *t*-tests were performed to evaluate the difference between the vibration sensations measured at the GT and MM.
8. Differences between the results elicited in the PNP group and those found in an age-matched subgroup of H (13 females and 9 males, aged ≥ 40 years (median 53, min 40, max 68)) were analyzed with independent samples *t*-tests.

## 3. Results

The mean durations of vibration sensation and the differences between the results elicited without and with socks are given in Table 1. The effect of wearing socks was found to be insignificant except that found on the MM in the H group, which was seen to have a small effect size upon further analysis with Cohen's *d* < 0.2  (*d* = 0.17). 

**Figure 2:**
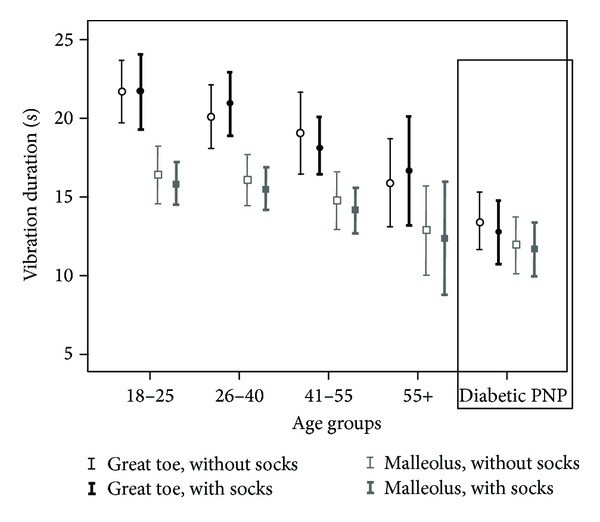
The duration of vibration sensation in different age groups of the healthy volunteers and the patients with diabetic polyneuropathy. Mean and 95% confidence interval values are given for the measurements without and with socks. Diabetic PNP: diabetic polyneuropathy.

Vibration sensation was found to decline with age in the H group for two measurement points and both conditions. Pearson's *R* in measurements without socks were −0.502 (*P* < 0.001) and −0.432 (*P* = 0.001), and with socks were −0.467 (*P* = 0.002) and −0.416 (*P* = 0.003), respectively (Figure 2). No correlation was found with height or weight in the H group.

The duration of vibration sensation was shorter at the MM as compared to GT in the H group (the mean difference was 4.3 s, *P* < 0.001). A smaller difference in the same direction was also observed in the PNP group (1.5 s mean difference, *P* = 0.012) (Figure 2). The vibration sensation of only one of the 50 participants in the H group, a 68-year-old male, measured less at the GT than at the MM, whereas 3 patients in the PNP group were found to have shorter vibration durations at the GT.

As compared to the age-matched subjects from the group H, the duration of vibration sensation in the PNP group had a significantly shorter vibration duration at the GT but not at the MM measurement points (*P* < 0.01 and *P* = 0.12, resp.). 

## 4. Discussion

The present study reveals that the mean durations of vibration sensation measured without and with socks are nearly the same; there is practically no difference between them in normal subjects and patients with diabetic polyneuropathy. Although a statistically significant reduction with socks was found in the measurements at the MM in group H, its effect size was small and the mean difference was less than one second. Since clinicians generally express the duration of vibration sensation by whole seconds, without using fractions, the size of the difference found at the MM does not seem important enough to affect the results of clinical examination. 

As far as we know, this is the first study on humans to investigate the perception of vibration over clothing. Other examples of clinical studies which investigated the effect of clothing during physical examination do exist, including the auscultation of lung sounds through thin clothing [5] and measurement of blood pressure through sleeves using the auscultatory method [6]. These studies all resemble each other in that the focus of interest is sounds and vibrations and the spectrums in question are also similar. Kraman showed that lung sound perceived by stethoscope was somewhat deteriorated by one or two layers of indoor clothing but this effect can be reduced by force on the stethoscope head [5]. Liebl et al. did not found statistically significant difference between sleeved and nonsleeved arm during manual auscultatory sphygmomanometric and automatic oscillometric blood pressure measurement [6]. An everyday experience was also found to be congruent with these results, that of a vibrating cell phone. It was observed that one is easily able to perceive the vibrations of a cell phone in her/his pocket through a significant amount of clothing. With this observation in mind, we measured the frequencies of incoming call vibrations of several models of cell phones and found that the range was 116 to 250 Hz. It is not surprising that this range includes the optimum frequency range for Pacinian corpuscles, namely, between 60 and 400 Hz [1]. All these data suggest that the interference from garments on vibration energy is little. With regard to the vibration of a cell phone in vibration sensing, the use of a pager device in its vibration mode was reported to be used for clinical vibration testing in an era when cellular phones were not so common [4].

The tuning fork is the most “readily available” instrument in daily clinical practice for measuring vibration sensation and is sufficiently reliable when compared to other techniques of measurement [7, 8]. Therefore, the tuning fork was the instrument of choice in this study. Several studies report the use of the Rydel-Seiffer graduated 64 Hz tuning fork because of its semiquantitative properties [7–9]. Although one was available at the installation phase of the present study, it was discarded because the semiquantitative scale of this device seemed to provide insufficient time resolution for our requirements. The other reason for discarding this tuning fork was that its 64 Hz was thought to be in the preferential frequency range of Meissner's corpuscles rather than the Pacinian afferents [1]. The physiology of Pacinian corpuscles may play a critical role in the sensation of vibration through clothing, due to their subcutaneous localization and wide field of perception [1, 10].

The shorter vibration durations at the MM than at the GT are an unexpected finding for clinicians familiar with axonal polyneuropathies, due to the distal to proximal progression of degeneration. It has been concluded that substantial tissue damping affects the tuning fork while taking measurements at the MM. This is probably caused by the solid mass of the tibia which is substantially larger than that of the toe bones beneath the GT measurement points. We think that the shorter vibration durations at the MM can be attributed to this phenomenon, which has been analyzed in detail in the article by Goldberg and Lindblom [11]. Mean vibration duration at the GT was higher than that at the MM even in the PNP group, although the difference was less. This may be due to the fact that this study involved only mild to moderate cases of diabetic PNP and excluded the more advanced cases in whom vibration sensation was measured as totally absent distally. It might be expected that, with more advanced disease, the GT measurement would have been able to exceed the MM in shortening of vibration sensation duration due to the distal axonopathy process.

In conclusion, this study shows that no practically significant difference arises from wearing socks when measuring vibration sensation with a tuning fork; any interference thus caused is negligibly small. This is true for the healthy population over a wide age range and also for patients within the clinical context of a diabetic polyneuropathy. However, this study does not aim to recommend measuring vibration sensation over socks. In the patients with chronic polyneuropathy, careful examination of the feet is crucial for the screening of ulcers and infections and leaving the socks in place during examination is strongly discouraged. Besides, the socks would still have to be removed in order to perform the other modalities of neurological examination. Bearing these in mind, we hope that the results of the present study will be considered as a clinical clue indicating one of the remarkable properties of vibration sensation. 

## Figures and Tables

**Table 1:** The duration of vibration sensation in the healthy volunteers and the patients with diabetic polyneuropathy.

Groups	Age (years) median (range)	Great toe (*s*, mean ± SD)				Medial malleolus (*s*, mean ± SD)			
		w/o socks	w/ socks	*D*	P*	w/o socks	w/ socks	*D*	P*
H 22 M, 28 F	37 (18–68)	19.4 ± 4.2	19.5 ± 4.2	−0.2 ± 1.67	*0.54 *	15.1 ± 3.3	14.6 ± 3.3	0.6 ± 1.62	**0.02**

PNP 5 M, 14 F	57 (32–72)	13.4 ± 3.8	12.7 ± 4.1	0.7 ± 2.13	*0.19 *	11.9 ± 3.8	11.7 ± 3.4	0.3 ± 1.82	*0.56 *

Abbreviations: H: healthy, PNP: patients with diabetic polyneuropathy, M: male, F: female, SD: standard deviation, w/: with, w/o: without, *D*: mean difference between measurements w/o socks and w/ socks. *Paired samples *t*-test was used.
